# Characterization of microbes and denitrifiers attached to two species of floating plants in the wetlands of Lake Taihu

**DOI:** 10.1371/journal.pone.0207443

**Published:** 2018-11-13

**Authors:** Bing Han, Songhe Zhang, Lisha Zhang, Kaihui Liu, Liying Yan, Peifang Wang, Chao Wang, Si Pang

**Affiliations:** Ministry of Education Key Laboratory of Integrated Regulation and Resource Development on Shallow Lakes, College of Environment, Hohai University, Nanjing, China; Free University of Bozen/Bolzano, ITALY

## Abstract

Biofilms are often observed at the solid-water interface. The leaves of many floating macrophytes have characteristics of both terrestrial plants and submerged macrophytes, because, in general, their upper and lower surfaces are exposed to air and water, respectively. However, little is known about the biofilms attached to floating plants. We investigated biofilms attached to the leaves, stems and roots of the floating plants *Nymphoides peltata* (in summer and winter) and *Trapa natans* (in summer) in the Gonghu Bay of Lake Taihu. Bacteria and algae were major components of the biofilm on the leaves of the two species of plants. In addition, 454 pyrosequencing analysis of bacterial 16S rRNA genes revealed that *Proteobacteria* was the dominant phylum, followed by *Bacteroidetes*, *Firmicutes*, *Chloroflexi*, *Acidobacteria*, and *Verrucomicrobia*. Cluster analysis showed that bacterial communities from the same plant source were clustered into the same group. A total of 677 genera were detected, and 47 genera were shared by all samples. Nitrifiers, including *Nitrosomonas*, *Nitrosococcus* and *Nitrospira* were detected in this study. Seven denitrifying genes (*napA*, *napG*, *nirS*, *nirK*, *cnorB*, *qnorB* and *nosZ*) were used to detect the abundance of denitrifiers. Genes *nirK*, *nirS cnorB* and *nosZ* were the four most abundant genes in all samples. Our results demonstrated that cultivation of floating plants in water column could enlarge the area for biofilm growth, and biofilms might play an important role in denitrification in eutrophic water.

## Introduction

Aquatic plants are widely distributed in a variety of rivers, lakes and other water bodies and are important parts of the natural landscape. Aquatic plant leaves typically do not experience desiccation stress and can therefore provide more ideal habitats for the survival of epiphytic microbes (known as biofilm or periphyton) [1,2]. Aquatic plants and biofilms are important parts of water self-purification systems in the water column and play important roles in the transformation of pollutants and the maintenance of ecological balance [3]. Therefore, aquatic plants are employed to treat wastewater or storm water [2].

An in-depth understanding of epiphytic microbes on aquatic plants is vital to understanding the important roles of coupled aquatic plant-epiphytes in freshwater ecosystems. To date, microbial communities have been investigated in various sources, including sediments [4], soil [5,6] and fish intestine [7]. Recently, He et al. [8] compared the diversity of epiphytic bacteria and surrounding bacterioplankton of a common submerged macrophyte using a high-throughput sequencing method. We further analyzed the distribution of microbes on the surface of submersed macrophytes [9]. Previous studies reported the influence of attached plant organs and seasonal variation to epiphytic microbes [9,10]. However, little is known about biofilms on the surface of floating plants.

Nitrogen is a key factor causing eutrophication. Wetland ecosystems maintain and improve water quality through denitrification, which is an important ecosystem service to counter global nitrogen pollution. In cattail-dominated sediments, denitrification rates are positively correlated with NH_4_^+^, sediment organic matter, reduced water levels, and cattail stand age [11]. Cultivation of free-floating plants such as *Eichhornia crassipes* can have a stimulating effect on the gaseous production of N_2_ by denitrification of eutrophic water [12]. The denitrification rate was the highest in sediments among floating-leaved macrophytes compared to bare sediment and submerged macrophyte-dominated sediment [13]. However, little is known about the distribution of denitrifiers in biofilms attached to floating plants.

We hypothesize that biofilms can form on the surface of floating plants, and many denitrifiers potentially have an important role in eutrophic water. Therefore, the aims of this study were to 1) confirm this hypothesis and 2) evaluate the similarity/difference in richness, diversity and composition of epiphytic microbes from different seasons and plant organs. Biofilm samples were collected from the surface of roots, stems and leaves of the floating plants *Nymphoides peltata* (in summer and winter) and *Trapa natans* (in summer) from sampling sites located in the Gonghu Bay of Lake Taihu. The quantity distribution of biofilm (bacteria and algae) and denitrification functional genes on the two species of aquatic plants was investigated. The community compositions of epiphytic bacteria and algae in biofilm samples were also analyzed.

## Materials and methods

### Biofilm sample collection

Lake Taihu is the third largest freshwater lake in China (Figure A in S1 File). Due to algal blooms and water pollution in recent decades, aquatic macrophytes have declined in most regions of Lake Taihu, except for Gonghu Bay, where two common floating macrophytes—*N*. *peltatum* and *T*. *natans*—are the dominant species. Therefore, plant samples of *N*. *peltatum* were collected in August (summer) and December (winter), 2013; *T*. *natans* was only sampled in August (summer) because it decays in December. During sampling, representative plant samples were collected by hand with gloves or with a stainless steel hook. Three biological replicates (more than fifty plants in each replicate) were collected from each sampling site for each species of plant.

Contaminants from the surrounding water or sediment were removed from the leaves, stems and roots of the collected plants (Figure B in S1 File) immediately using distilled water, and the plant samples were stored in aseptic plastic bags. For each sampling site, 1 L of surface water (50 cm under water surface) surrounding the *N*. *peltatum* and *T*. *natans* plants was collected for physicochemical analyses. All plant samples were stored at 4°C and transferred to the laboratory within 3–5 h. Water quality parameters are provided in Table A in S1 File.

### Detachment of biofilm microbes

Biofilm microbes were detached from approximately 20 g plant samples into a sterile 500-mL polyethylene tube using 400 mL cold phosphate buffer solution (PBS, pH 7.2) and small glass beads (1-mm diameter). The samples were ultra-sonicated for 3 min, shaken for 30 min and subsequently ultra-sonicated for 3 min. Plant debris was removed from the detachments by passage through a 100-mm diameter sieve (150 μm pore size). Approximately 5 mL of the detached sample was fixed in a final concentration of 2% formaldehyde solution for epiphytic bacteria count. Approximately 5 drops of Lugol’s reagent were added to 5 mL detached sample solution for epiphytic algae analysis. For DNA extraction, approximately 400 mL of solution was filtered using 0.22-μm membranes, and the filters were stored at -80°C. Leaf surface area was measured using a method previously described [14].

### Dying count of bacteria and microscope examination of algae

Approximately 100 μL microbe sample fixed with formaldehyde was mixed with 700 μL 10 mg L^-1^ 4',6-diamidino-2-phenylindole (DAPI) and incubated for 30 min at room temperature. The mixtures stained with DAPI were filtered onto dark polycarbonate filters (0.22-μm pore size, 25-mm diameter) using a vacuum pump. The number of epiphytic microbial cells was recorded using a fluorescence microscope (Axioskop 40, Carl Zeiss, Germany). Algae were enumerated using a blood cell counting chamber. The genus of observed algae was distinguished according to the algae taxonomy and the number was recorded and analyzed. Samples were analyzed in triplicates.

### Scanning electron microscope (SEM) analysis

Leaf samples were cut into approximately 0.5 cm ×0.5 cm squares and incubated in 2.5% glutaraldehyde solution for 12 h and 1% OsO_4_ solution for 3 h. After three rinses with 0.1 M sodium phosphate buffer solution (15 min each time), leaf samples were dehydrated in a serial concentrations of ethanol (in sequence: 30, 50, 70, 80 and 90% ethanol, and 15 min for each concentration), followed by 100% ethanol three times for 15 min each time. The dehydrated samples were dried in a freeze-drier for approximately 12 h. The gold-sprayed leaf samples were visualized using a scanning electron microscope (S-4800, Hitachi, Japan).

### 454 pyrosequencing and data processing

Biofilm DNA was extracted from approximately 0.25 g biofilm sample using a PowerBiofilm DNA Isolation kit (MoBio Laboratories, U.S.) according to the manufacturer’s protocols. Three extracted DNA solutions from the same sample were used for PCR and 454 pyrosequencing. Primer sets 342F (5’-CTACGGGGGGCAGCAG-3’) and 806R (5’-GGACTACCGGGGTATCT-3’) were used to amplify partial DNA sequences of bacterial 16S ribosomal RNA genes [15]. Briefly, a 20 μL mixture was prepared with 4 μL 5 × FastPfu Buffer, 2 μL 2.5 mM dNTPs, 0.8 μL of each primer (5 μM), 0.4 μL FastPfu Polymerase, 10 ng template DNA and suitable ddH_2_O. PCR reactions were performed at 95°C for 3 min, followed by 25 cycles at 95°C for 30 s, 55°C for 30 s, and 72°C for 30 s and a final extension at 72°C for 5 min. The PCR products were purified using an AxyPrep DNA Gel Extraction Kit (Axygen Biosciences, U.S.) and quantified using spectrometry (NanoDrop-1000, Thermo Scientific, U.S.). Amplicons were used for pyrosequencing on a Roche 454 GS FLX+ Titanium platform (Roche 454 Life Sciences, U.S.) according to standard protocols. The low-quality sequences were removed using QIIME (version 1.17). Operational Taxonomic Units (OTUs) were clustered with a 97% similarity cutoff using UPARSE (version 7.1 http://drive5.com/uparse/), and chimeric sequences were identified. RDP Classifier (http://rdp.cme.msu.edu/) was used to identify the phylogenetic affiliation of each 16S rRNA gene sequence against the Silva 16S rRNA database at a confidence threshold of 70% [16]. OTUs that reached the 97% similarity level were used for diversity, richness (Ace), Good’s coverage, and rarefaction curve analysis using Mothur (version 1.5.0 http://www.mothur.org/wiki/Schloss_SOP#Alpha_diversity) [17].

### Detection of denitrification functional genes

The abundance of denitrification genes, including periplasmic nitrate reductase (*napA*), membrane-bound nitrate reductase (*narG*), nitrite reductase (*nirS* and *nirk*), nitric oxide reductase (*cnorB* and *qnorB*) and nitrous oxide reductase (*nosZ*), were detected using quantitative polymerase chain reaction (qPCR). A 20-μL reaction mixture was prepared with 1 μL template DNA, 10 μL SYBR Green I PCR master mix (Bio-Rad, U.S.), 1 μL forward and reverse primers and 7 μL sterile water. The qPCR was conducted using a CFX96 Touch real-time PCR Detection System (Bio-Rad, U.S.) according to our previous study [9]. Each qPCR amplification for each gene comprised a total of 40 cycles. The primer sequences are provided in Table B in S1 File. Three biological replicates were used.

### Statistical analysis

Bacterial and algal densities were analyzed using one-way ANOVA and Tukey’s test using the software SPSS (version 19.0). Cluster analysis based on Bray–Curtis index and principal component analysis were conducted with the software PAST (version 3.10) and Canoco (version 4.56), respectively. Bar plots of the main phyla were generated to compare the difference between samples. Heatmap of algal genera of was employed to depict similarity and dissimilarity among algae communities with the software HemI (version 1.0, http://hemi.biocuckoo.org). Two-way ANOVA analysis was employed to compare the richness of denitrifying genes from different samples.

## Results and discussion

### Bacteria and algae in biofilms attached to floating plants

**Fig 1:**
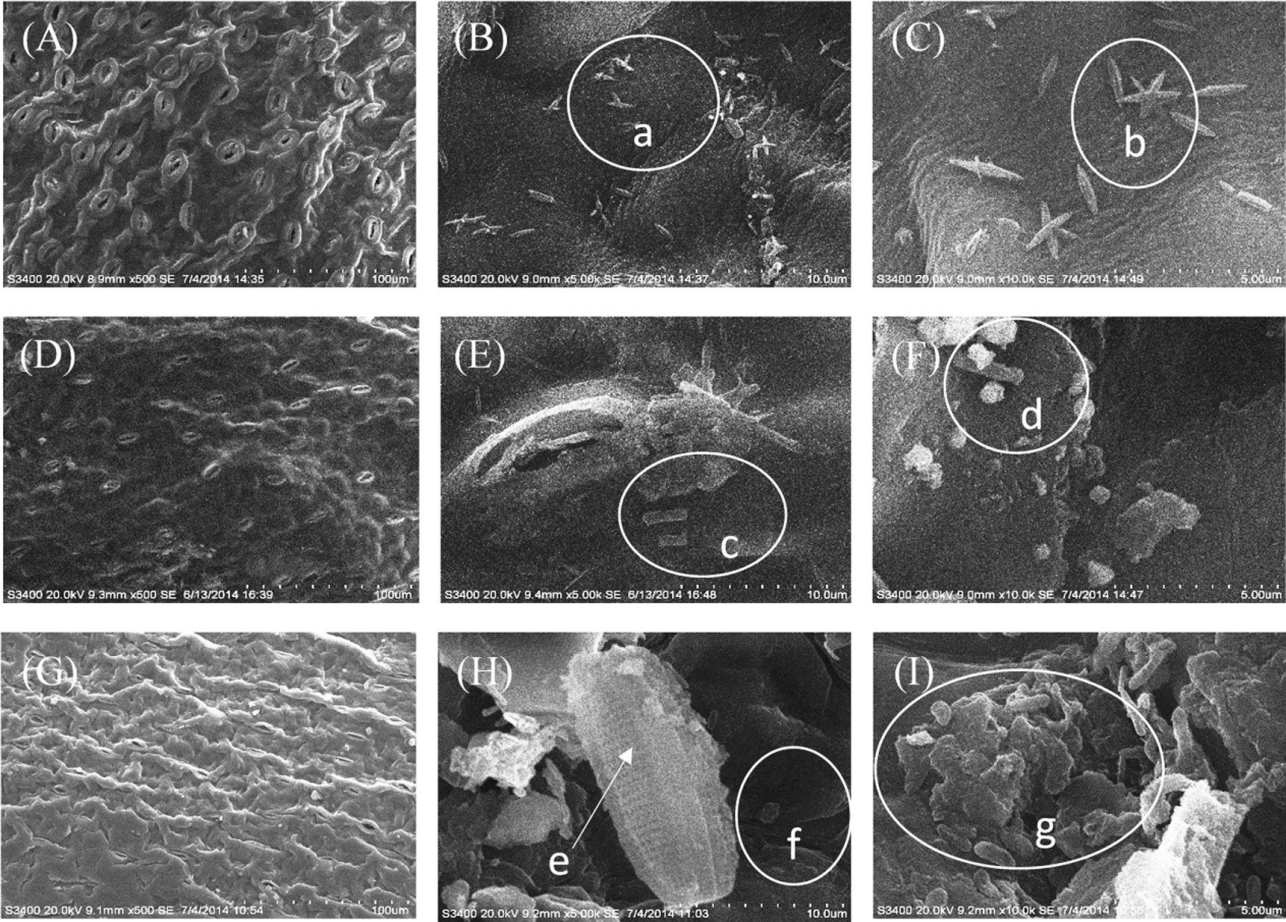
**Scanning electron microscope images of the lower leaf surface of *N*. *peltatum* (A-C, summer sample; D-F, winter sample) and *T*. *natans* (G-I, summer sample).** (A, D and G, 500x; B, E and H, 5,000x; C, F and I, 10,000x). a, b, c and d, bacilli; e, algae; f, cocci; and g, microbe aggregation.

The bacterial community in the phyllosphere has been intensively studied for terrestrial plants and has received considerable attention in recent years [18]. The leaves of many floating macrophytes have characteristics of both terrestrial plants and submerged macrophytes, because, in general, their upper and lower surfaces are exposed to air and water, respectively. However, little information is known about the bacteria community in the phyllosphere of floating macrophytes. As revealed by SEM images, abundant microbes were observed on the lower leaf surface of *N*. *peltatum* and *T*. *natans* (Fig 1) but almost no microbes were observed on the upper surface (Figure C in S1 File). The upper surface exposed to atmosphere can be influenced by ultraviolet rays and desiccation [19,20], which inhibits the growth of microbes. Abundant bacteria and algae were observed, and the bacteria always inhabited the junctions of epidermal cells (Fig 1). Previous studies have also shown that bacteria and algae are major components of epiphytic microbes on submerged macrophytes [21–23].

**Fig 2:**
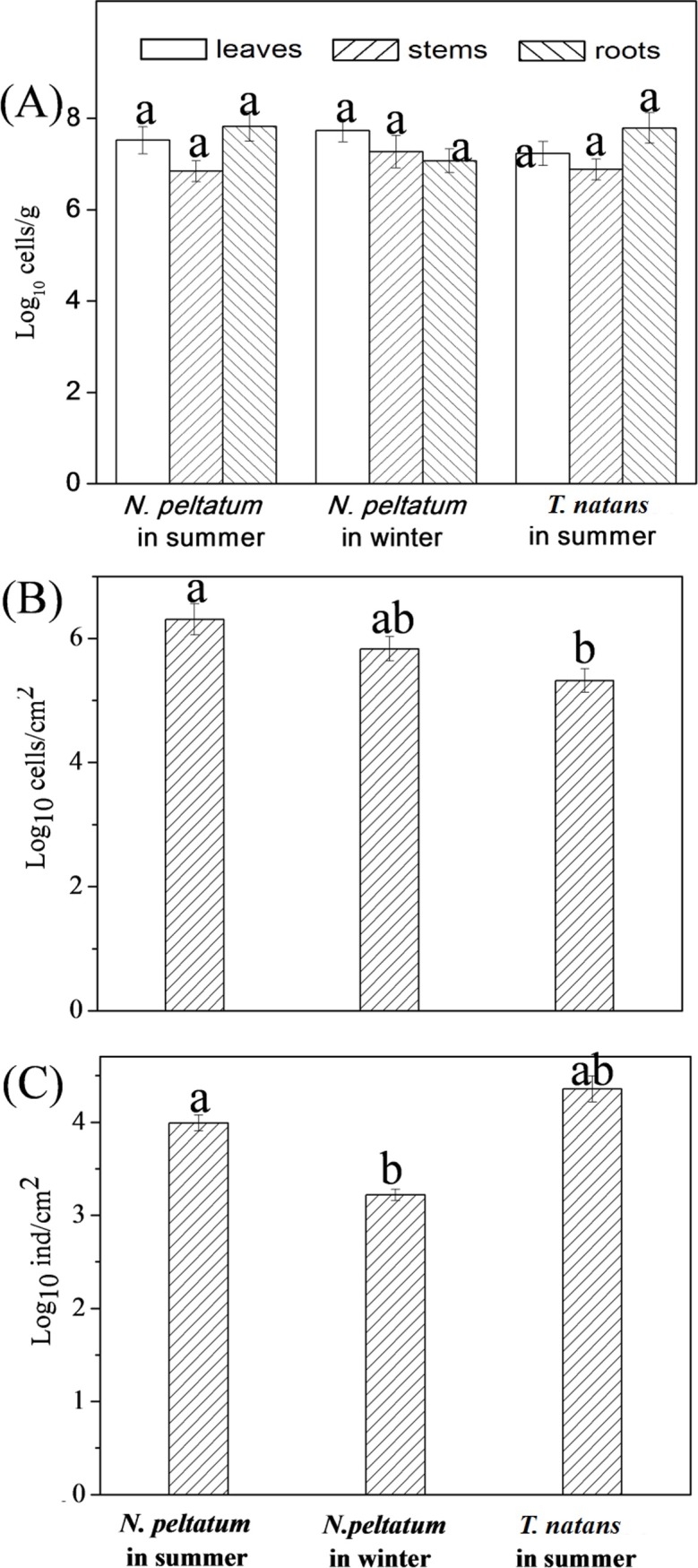
**Total densities of bacteria and algae on the leaf, stem and root samples (A, expressed as log**_**10**_ **g**^**-1**^ **dry mass) and densities of bacteria (B, expressed as log**_**10**_ **cells cm**^**-2**^**) and algae (C, expressed as log**_**10**_ **cells cm**^**-2**^**) on the three leaf samples.** Different small letters (a, b or ab) indicate significant differences among samples at *p* < 0.05 (Tukey’s test).

No significant differences in the total densities of bacteria and algae (10^6^−10^8^ cells g^-1^ dry mass) were detected among samples from the leaves, stems and roots of the same species of the floating plant (Fig 2A). However, significant differences (*p* < 0.05) in the densities of bacteria/algae (cells cm^-2^) were detected among the three leaf samples. The densities of epiphytic bacteria on the leaf surface were in the following order: *N*. *peltatum* in summer (2.03±0.08 ×10^6^ cells cm^-2^), *N*. *peltatum* in winter (6.80±0.23 ×10^5^ cells cm^-2^) and *T*. *natans* in summer (2.10±0.08 ×10^5^cells cm^-2^) (Fig 2B). For epiphytic algae (Fig 2C), the densities on the leaf surface were in the following order: *T*. *natans* in summer (2.28±0.07 ×10^4^ cells cm^-2^), *N*. *peltatum* in summer (9.79±0.21 ×10^3^ cells cm^-2^) and *N*. *peltatum* in winter (1.66±0.03 ×10^3^ cells cm^-2^). Similar levels of cell densities were found on the submerged plants *Chara aspera* from brackish water and *Myriophyllum spicatum* in fresh water [23]. Previous study reported that larger bacteria densities (cells g^-1^ dry mass) were usually found at rhizosphere than at phyllosphere (including leaves, stems, blossoms and fruits) of the rooted terrestrial plants [24]. The difference might be ascribed to the floating lifestyle of the two species of plants in the present study, with the roots suspending in the water column rather than in sediments. Surface characteristic and allelopathy exudates of the aquatic plants were reported to be the main biotic factors affecting the epiphytic biofilm formation [25]. In addition, low temperature might inhibit the growth of microbes in winter.

Based on the SEM images (Fig 1) and densities of bacteria and algae (Fig 2), we concluded that the densities of bacteria were higher than that of algae in biofilms on the leaves of the two species of plants. These differences may be ascribed to the natural characteristics of bacteria and algae as well as the impact of the hosts, as the microbial epiphytes are influenced by sunlight, water quality and plant metabolism [19,20]. The bacteria are generally smaller than algae (except for cyanobacteria); therefore, it is easier for bacteria to adhere to the substrate surface than algae [26]. Metabolites secreted by aquatic plants may be another reason for why there are more epiphytic bacteria than epiphytic algae [27,28]. Previous reports [22] showed that seasonal variations and other environmental factors had a greater influence on epiphytic bacteria than the growth status of aquatic plants.

### Composition of epiphytic algae on the leaves

In total, five algae phyla were detected in the three leaf samples (Figure S4 and Table C in S1 File), including *Chlorophyta*, *Cyanophyta*, *Bacillariophyta*, *Xanthophyta* and *Cryptophyta*. In summer, *Chlorophyta* was the most abundant phylum on plants of *N*. *peltatum* (33.44%) and *T*. *natans* (34.75%), followed by *Bacillariophyta* and *Cyanophyta*. By contrast, *Bacillariophyta* was the dominant phylum in leaf samples of *N*. *peltatum* in winter (39.35%), followed by *Chlorophyta* (35.72%) (Figure D in S1 File). Overall, 25 algae genera were detected, and 17, 16 and 19 genera were attached to the leaves of *N*. *peltatum* in summer, *N*. *peltatum* in winter and *T*. *natans* in summer, respectively. Shannon indices of epiphytic algae on the leaves of *N*. *peltatum* in summer, *N*. *peltatum* in winter and *T*. *natans* in summer were 2.65±0.16, 2.68±0.16 and 2.72±0.19, respectively. Cluster analysis revealed that the algae community structure on the leaves of *N*. *peltatum* in summer was more similar to that of the same species in winter than to that of the *T*. *natans* in summer (Figure D in S1 File). Most algae can inhabit floating plants in summer and winter (Table C in S1 File). Previous study also found that most of the epiphytic algae on aquatic (emergent and submerged) plants in shallow lakes belonged to *Chlorophyta*, *Cyanophyta* and *Bacillariophyta*, and the main influence factors were attributed to the host architecture and allelopathy [29]. Epiphytic algal communities may also have been selected for their relative insensitivity to alleleopathic compounds [30]. A recent report [8] showed that algae in biofilms attached to submersed macrophytes have competitive advantages compared to planktonic algae in the acquisition of nutrients from the water and from plant tissues.

### Diversity of bacteria communities

After filtering, 101,574 high-quality reads from nine samples (8,389 to 15,922 reads) were used for further analysis (Table D in S1 File). The OTU numbers ranged from 221 (13,829 filtered reads, the stems of *T*. *natans* in summer) to 1620 (15,922 filtered reads, the roots of *N*. *peltatum* in summer). Based on the OTU numbers, Chao values and Shannon values, the diversities of bacteria communities from *N*. *peltatum* sources were in the following order: leaf < stem < root both in summer and winter. By contrast, for *T*. *natans* in summer, the diversity of bacteria communities from stem sources was lower than that from leaf and root sources (Table D in S1 File). This result may be due to the special shape of the stems of *T*. *natans*. Our previous study [3] showed that bacterial community diversity was related to the species of macrophyte according to different leaf shapes. Although the number of new bacterial 16S rRNA gene sequences increased with increased sequence number (Figure E in S1 File), the Shannon-Wiener values reached a stable level after the number of bacterial 16S rRNA gene sequences reached 2,000 (Figure E in S1 File). This result suggested that the numbers of reads (Table D in S1 File) obtained in this study were sufficient to analyze diversity at the bacterial community level.

**Fig 3:**
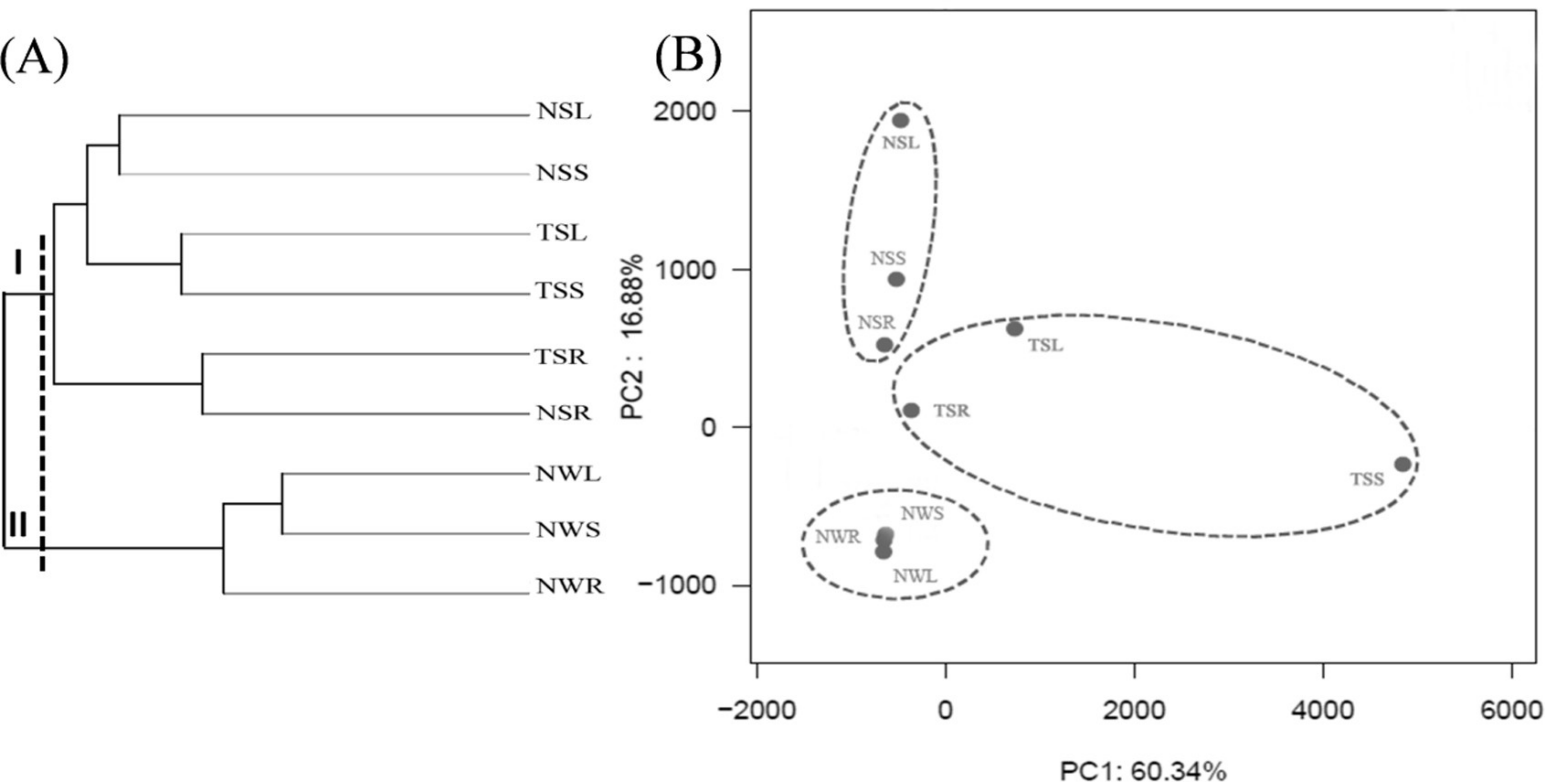
Cluster analysis (based on the Bray-Curtis algorithm) and principal component analysis of all samples at genus level. Samples attached to leaves (NSL), stems (NSS) and roots (NSR) of *N*. *peltatum* in summer, to leaves (NWL), stems (NWS) and roots (NWR) of *N*. *peltatum* in winter, and to leaves (TSL), stems (TSS) and roots (TSR) of *T*. *natans* in summer.

Cluster analysis was employed to evaluate the similarities at OTU level (Fig 3A). Samples collected from *N*. *peltatum* sources in summer and winter were clustered into group I and group II, respectively. Cai et al. [22] reported no significant intra-lake heterogeneity in community structure from submerged macrophyte *Potamogeton malaianus* in Lake Taihu, but the temporal heterogeneity of epiphytic microbes was significant. These researchers suggested that alterations in community structure were linked to the growth state of submerged macrophytes and water temperature. Bacteria communities from the leaf and stem sources of the same species of plant were clustered together, whereas bacteria communities from the root sources of the two species of plants in summer were more similar to each other than to other samples. Similar to our results, bacterial communities in leaf and stem samples differed from root samples for terrestrial plants [10]. Host-specified bacteria community was found in epiphytic microbes on submerged plants in fresh water [21] and rhizosphere microbes on rooted terrestrial plants [24]. To better understand the relationships among bacterial community compositions, principal component analysis was performed based on the composition of OTUs (with 97% similarity cutoff), and two main components were generated (Fig 3B). The total variances of components 1 and 2 were 60.34% and 16.88%, respectively.

### Characterization of bacterial communities

**Fig 4:**
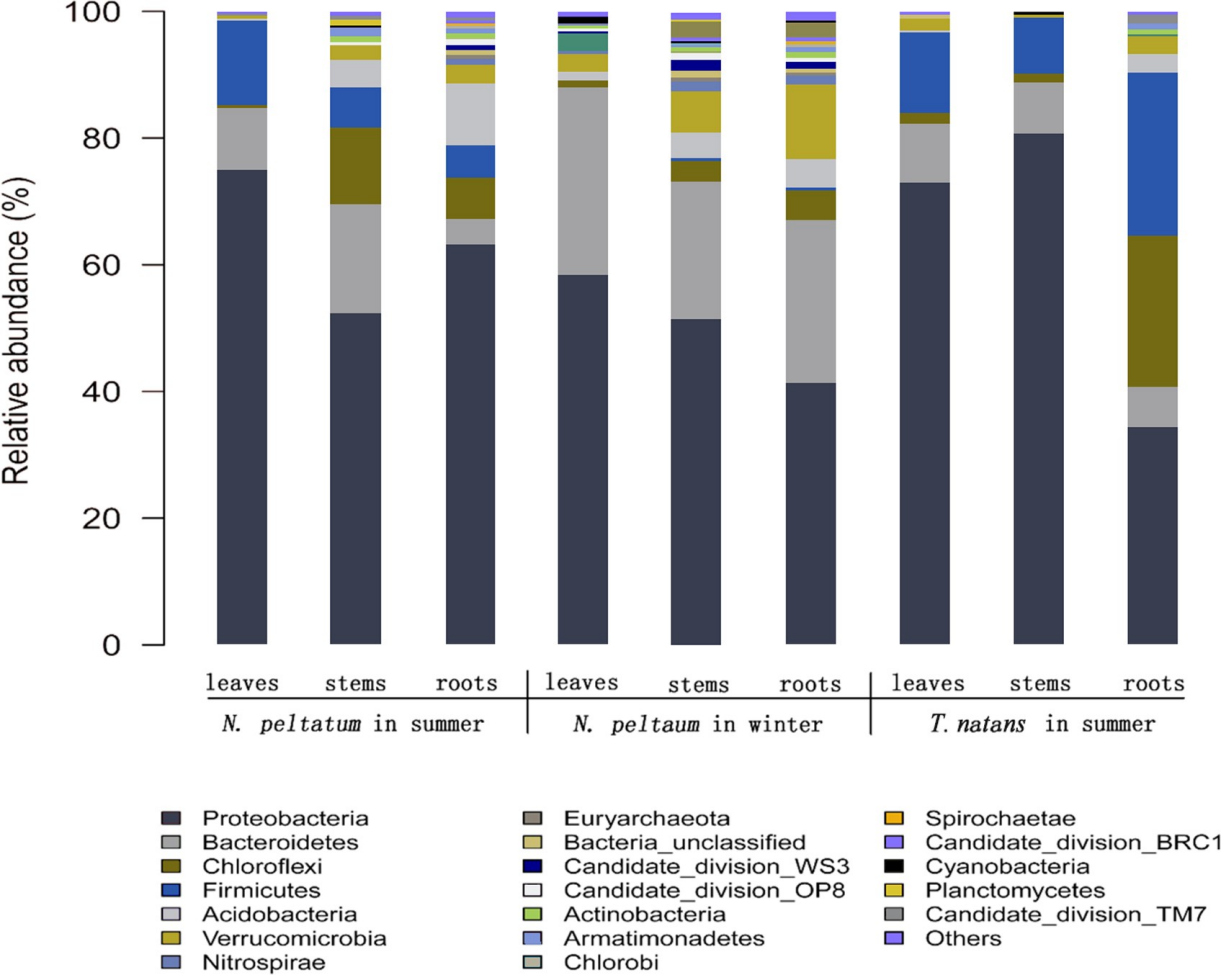
Bacterial community structures at phylum level for all samples. Samples attached to leaves (NSL), stems (NSS) and roots (NSR) of *N*. *peltatum* in summer, to leaves (NWL), stems (NWS) and roots (NWR) of *N*. *peltatum* in winter, and to leaves (TSL), stems (TSS) and roots (TSR) of *T*. *natans* in summer. The abundance of phyla making up less than 1% of total high-quality sequences were classified as ‘other’ in all samples.

A total of 38 phyla were detected in all 9 samples (Fig 4 and Table E in S1 File), and 33 and 36 phyla were detected in samples from *N*. *peltatum* in summer and winter, respectively. By contrast, only 22 phyla were detected in *T*. *natans* in summer. *Proteobacteria* (34.54–80.83%) was the most abundant phylum in all samples, followed by *Bacteroidetes* (3.93–30.16%), *Firmicutes* (0.02–25.71%), *Chloroflexi* (0.57–23.87%), *Acidobacteria* (0.25–9.9%), and *Verrucomicrobia* (0.36–11.82%). Similar to our results, members of *Proteobacteria*, *Firmicutes*, *Actinobacteria*, *Bacteroidetes* and *Chloroflexi* were also dominant in various sources, including leaves of submerged plants [21], leaves and roots of the terrestrial plants *Arabidopsis thaliana* [31] and *Stellera chamaejasme* [10], and sediments from Lake Taihu [32].

Classes *Alphaproteobacteria*, *Betaproteobacteria* and *Gammaproteobacteria* dominated in the phylum *Proteobacteria* in all samples, with the exception of *Betaproteobacteria* (< 1%) in the stems of *T*. *natans* in summer, as indicated by the α-diversity values. *Deltaproteobacteria* was abundant in sources from the roots of *N*. *peltatum* in summer and winter, the stems and leaves of *N*. *peltatum* in winter but occurred at low frequency (< 1%) in other samples. The classes *Alphaproteobacteria*, *Betaproteobacteria*, *Deltaproteobacteria* and *Gammaproteobacteria* were also dominant in biofilms from submerged aquatic plants [8,21,23] and in the sediment of Lake Taihu [32]. Using FISH analysis, Fuchs and Glockner [33] found that *Betaproteobacteria* occurred almost exclusively in fresh water but not in saline habitats, whereas *Alphaproteobacteria* was more abundant in marine than in freshwater samples. *Alphaproteobacteria* was the dominant class within *Proteobacteria* from saline sludge samples, whereas *Betaproteobacteria* was the dominant class, followed by *Alphaproteobacteria*, *Gammaproteobacteria* and *Deltaproteobacteria* [34]. It should be noted that within the phylum *Proteobacteria*, *Alphaproteobacteria* was the dominant class in samples from the roots and leaves of *T*. *natans* and the stems of *N*. *peltatum* in summer, whereas *Betaproteobacteria* dominated in samples from the stems, roots and leaves of *N*. *peltatum* in winter, with *Gammaproteobacteria* in samples from the roots and leaves of *N*. *peltatum* and the stems of *T*. *natans* in summer. *Epsilonproteobacteria* was not detected or occurred at less than 0.2% in all samples (Table E in S1 File). Therefore, the successions of bacterial communities in biofilms should be further studied in the life cycle of aquatic macrophytes.

A total of 36 abundant orders (abundance > 1% in at least one sample) accounted for 81.9–98.7% of filtered reads from all samples at order level (Table F in S1 File). Among the 36 abundant orders, *Bacillales*, *Burkholderiales*, *Chthoniobacterales*, *Enterobacteriales*, *Flavobacteriales*, *Pseudomonadales*, *Rhizobiales*, *Rhodobacterales*, *Sphingomonadales* and *Xanthomonadales* were detected in at least five samples. It should be noted that orders *Chromatiales*, *Cytophagales*, *Methylophilales*, *Rickettsiales* and *Verrucomicrobiales* were only abundant in samples from *N*. *peltatum* in winter. The 28 families, belonging to the top 10 abundant families in at least one sample, occurred in 65.4–96.6% of reads from 9 samples (Table G in S1 File). *Caldilineaceae*, *Comamonadaceae*, *Flavobacteriaceae*, *Rhodobacteraceae*, *Sphingomonadaceae* and unclassified *Sphingomonadales* were abundant orders in at least four samples. *Caldilineaceae*, *Enterobacteriaceae*, *Flavobacteriaceae Methylophilaceae*, *Moraxellaceae* and *Sphingomonadaceae* accounted for >10% of the classified sequences in at least one sample. A recent study on bacterioplankton showed that *Sphingomonadales* was found to be more related to NO_3_^-^, whereas *Methylophilales* was positively related to NO_2_^-^ and PO_4_^3-^ [35]. The results were consistent with our findings in the present study. As shown in Table A in S1 File, higher concentrations of NO_3_^-^ were detected in summer whereas higher concentrations of NO_2_^-^ and PO_4_^3-^ were determined in winter. The results showed that the seasonal variation of nutrients might affect the epiphytic bacteria composition on floating plants. Seasonal succession of rhizosphere bacterial community of the terrestrial grass was attributed to the seasonal dynamics of available nutrients [36].

In all 9 samples, a total of 677 genera were detected, and the numbers of genera ranged from 129 to 474 (Table H in S1 File). Forty-seven genera (accounting for 63.67% of the total high-quality sequences) were shared by all nine samples. A total of 40 genera belonged to the top 10 abundant genera in at least one sample, and among them, the genera *Enterobacteriaceae unclassified*, *Pseudomonas*, *Chryseobacterium*, *Rhodobacter*, *Sphingomonadales_unclassified*, *Exiguobacterium*, *Comamonadaceae_unclassified* and *Acinetobacter* were detected in at least 5 samples. The genera *Azospirillum*, *Cloacibacterium*, *Enterobacter*, *unclassified Enterobacteriaceae*, *Exiguobacterium* and *uncultured Veillonellaceae* were markedly higher only in samples collected in summer than that in winter, whereas reverse trends were observed for the genera *Flavobacterium*, *Methylotenera*, *Albidiferax*, *Duganella*, *Janthinobacterium* and *Pseudorhodoferax*. *Acinetobacter* accounted for 6.88–60.99% of classified sequences in biofilms from *T*. *natans* in summer, but were detected at low abundance (0.06–1.66%) in samples from *N*. *peltatum* in winter and summer. *Novosphingobium* occurred at higher percentages (2.54–4.65%) in samples from *N*. *peltatum* in summer than those from *N*. *peltatum* in winter (0.52–0.82%) and *T*. *natans* (0.27–1.49%) in summer. The differences in biofilms may be ascribed to the temperature or status of plant growth [37, 38].

Many potential pathogenic genera, including *Aeromonas*, *Clostridium*, *Flavobacterium*, *Pseudomonas* and *Vibrio* [39] were abundant (> 1%) in many of these samples. *Aeromonas*, *Acinetobacter*, *Lysinibacillus*, and *Lactococcus*, suggested as probiotics and potential pathogens of gibel carp [7], were also detected in this study. Although 265 rare genera (occurring in no more than two samples) occupied approximately 1.15% of the total high-quality sequences, many of these genera have important ecological roles. For example, nitrifiers including *Nitrosomonas*, *Nitrosococcus* and *Nitrospira* usually represented less than 2% of the total bacterial population in samples investigated in this study. Zhang et al. [34] suggested that although nitrifiers normally occur in less than 1% of the total bacterial community in the activated sludge of full-scale WWTPs, they are extremely important for nitrogen removal. Overall, the floating plants provide diverse habitats for epiphytic bacteria, and the bacteria diversity are promoted by the community diversity of floating plants.

### Abundance of denitrification genes

Aquatic macrophytes have an important role in aquatic ecosystems. Like terrestrial plants in the rhizosphere of benthic vegetation, denitrification coupled to nitrification can inhibit or stimulate nitrogen loss in agricultural watersheds [40]. In addition, many aquatic macrophytes can provide multiple interfaces for the growth of denitrifying bacteria [3], and in agricultural watersheds, nitrogen removal by macrophyte stands were less than by nitrogen-related processes in microbes [40]. In nitrogen-related processes, denitrifying bacteria harboring metalloenzymes (e.g. nitrate reductase, nitrite reductase, nitric oxide reductase and nitrous oxide reductase, etc.) play important roles in the nitrogen cycles of various ecosystems. However, there are only a few investigations of the occurrence of denitrifiers in epiphytic communities in natural environments [34].

Many denitrifying bacteria harboring *narG* and/or *napA* can reduce NO_3_^-^ to NO_2_^-^ in the denitrification process [41]. The relative abundance of *napA* and *napG* ranged from approximately 18 (the roots of *N*. *peltatum* in summer) to 740 (the leaves of *N*. *peltatum* in summer) copies μg^-1^ DNA and from 10 (the roots of *N*. *peltatum* in summer) to 378 (the leaves of *N*. *peltatum* in winter) copies μg^-1^ DNA, respectively. Similarly, the copy numbers of *napA* genes were generally higher than that of *napG* in estuarine sediments [41]. There is a higher diversity of *narG* in association with macrophyte rhizospheres (*Littorella uniflora* and *Myriophyllum alterniflorum*) compared to that in unvegetated sediment [42]. Genes encoding *nirS* or *nirK* are involved in the reduction of NO_2_^-^ to NO [34]. The *nir*S and *nirK* genes were detected in all samples. Copy numbers of *nirS* in biofilm DNA samples from leaves and stems of *N*. *peltatum* were higher than those from roots, whereas for *T*. *natans* the highest densities of *nirS* were observed in root samples (Table I in S1 File). Denitrification rates and abundance values of nitrogen-cycling genes (*nirS* and *nirK*) in vegetated sediments were higher than those in bare sediments [43].

**Fig 5:**
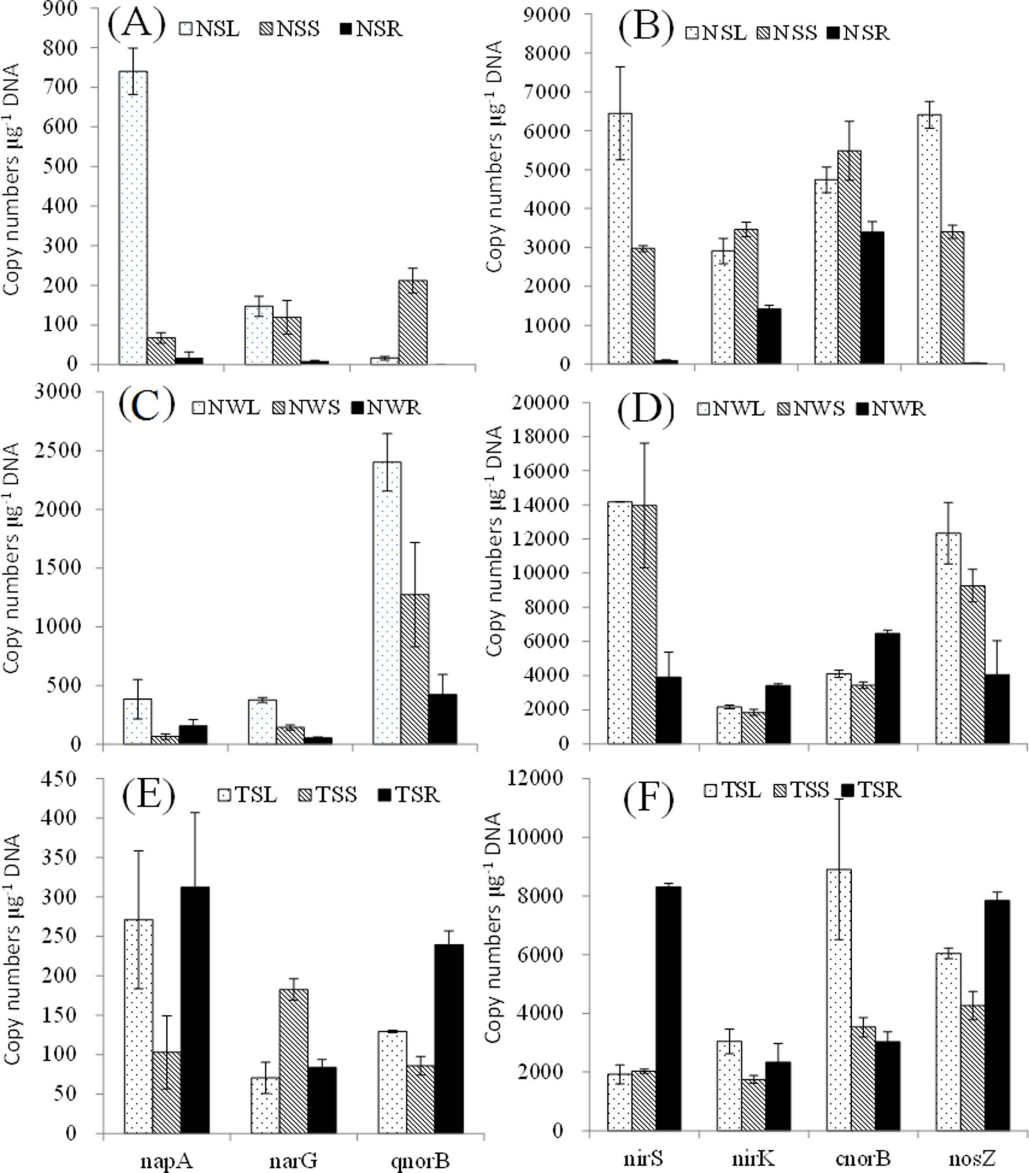
Abundance of denitrification genes in biofilm. A and B: samples attached to leaves (NSL), stems (NSS) and roots (NSR) of *N*. *peltatum* in summer; C and D: samples attached to leaves (NWL); stems (NWS) and roots (NWR) of *N*. *peltatum* in winter; E and F: samples attached to leaves (TSL), stems (TSS) and roots (TSR) of *T*. *natans* in summer.

Nitric oxide reductase (*cnorB* and *qnorB*) converts NO to N_2_O. The abundance of *cnorB* (ranging from 186 to 12,341 copies μg^-1^ DNA) was higher than that of *qnorB* (ranging from undetectable to 2,400 copies μg^-1^ DNA) for the same sample (Fig 5). Genes encoding the protein *cnorB* catalyze nitric oxide reduction on the outside of the cytoplasmic membrane and the *cnorB* gene has been detected by PCR in aerobic denitrifiers [34]. However, in this study, the abundance of the *cnorB* gene did not vary obviously among all biofilm samples, and the *qnorB* gene showed low abundance in samples collected in winter (Table I in S1 File). Nitrous oxide reductase *nosZ* is associated with the reduction of N_2_O to N_2_ and plays key roles in the last step of denitrification [34]. Copy numbers of the *nosZ* gene were detected in all samples except for the roots of *N*. *peltatum* in summer.

In seven denitrification genes, *nosZ*, *cnorB*, *nirK* and *nirS* were dominant in all samples (Fig 5), indicating that bacteria harboring these genes have an important role in denitrification. Our previous study showed that *nirS*, *nirK* and *cnorB* were also dominant denitrifying genes on the leaves of submerged macrophytes [3]. A previous report [12] showed that the presence of *E*. *crassipes* significantly promoted the abundance of *nosZ*, *nirK* and *nirS* in the water and indicated that cultivation of *E*. *crassipes* could have a stimulating effect on the gaseous production of N_2_ by denitrification in eutrophic water. The abundance of denitrifiers can be affected by aquatic macrophyte species, nutrient level and environmental parameters [3]. Many of these denitrifying genes can be expressed under aerobic conditions and have important roles in denitrification [44]. The oxygen level in biofilms can be affected by respiratory consumption and photosynthetic process in the dense stands of aquatic vegetation regions, and this might result in a shift from aerobic to anaerobic bacterial respiration at night, which is beneficial for denitrification [45].

## Conclusions

In conclusion, the lower surface of floating plants can be colonized by microbes, and bacteria and algae are major components of epiphytic microbes. Microbial densities did not vary significantly among different organs for the same species of plant (expressed as cells g^-1^), but significant differences in bacterial and algal density (expressed as cells cm^-2^) were found among the three leaf samples. *Proteobacteria* was the dominant phylum in nine samples, followed by *Bacteroidetes*, *Firmicutes*, *Chloroflexi*, *Acidobacteria* and *Verrucomicrobia*. Bacterial communities showed high similarities among sources from various parts of the same species of plant at the same sampling site. Seven denitrification genes were detected in all biofilm samples, and the genes *nosZ*, *cnorB*, *nirK* and *nirS* were the four most abundant genes in all samples. These results provide useful information for understanding the microbes on the surface of floating plants in wetlands. However, further investigation is needed to determine the ecological role of these microbes.

## Supporting information

S1 File This file contains figures A-E and tables A-I. (DOC)
