# Supplementary material for: Characterization of microbes and denitrifiers attached to two species of floating plants in the wetlands of Lake Taihu
Source: PLoS One. 2018 Nov 13;13(11):e0207443. doi: 10.1371/journal.pone.0207443 (PMC6233912; doi:10.1371/journal.pone.0207443)
Supplement: S1 File — (DOC) [file pone.0207443.s001.doc]

Characterization of microbes and denitrifiers attached to two species of floating plants in the wetlands of Lake Taihu

Bing Han^1^, Songhe Zhang^1,*^, Lisha Zhang^1^, Kaihui Liu^1^, Liying Yan^1^, Peifang Wang^1,*^, Chao Wang^1^, Si Pang^1^

^1^Ministry of Education Key Laboratory of Integrated Regulation and Resource Development on Shallow Lakes, College of Environment, Hohai University, Nanjing 210098, China

^*^Corresponding authors

Email: shzhang@hhu.edu.cn; pfwang2005@hhu.edu.cn

# Methods and materials

## Nutrient levels in surface water

Approximately 200 mL of surface water sample was filtered (0.22 μm pore size, 25 mm diameter) using a vacuum pump. The filtered water was used to determine the concentrations of total phosphorous (TP), total nitrogen (TN), nitrate nitrogen (NO_3_^－^-N), nitrite nitrogen (NO_2_^－^-N) and ammonia nitrogen (NH_4_^+^-N). TP, TN, NO_3_^－^-N, NO_2_^－^-N and NH_4_^+^-N in surface water were detected by a continuous colorimetric flow analysis (Auto Analyzer 3, Germany). The permanganate index (COD_Mn_) was monitored by titration.

# Results

There were some variances in primary water quality index between August (summer) and December (winter) in Lake Taihu (Table A in S1 File). We observed that the concentrations of TP (0.17±0.01 mg L^-1^), TN (4.52±0.21 mg L^-1^) and NH_4_^+^-N (1.56±0.08 mg L^-1^) in winter were higher than that in summer (TP, 0.13±0.01 mg L^-1^; TN, 3.83±0.18 mg L^-1^; and NH_4_^+^-N, 0.82±0.04 mg L^-1^). However, the concentrations of NO_3_^－^-N (2.79±0.13 mg L^-1^), COD_Mn_ (3.56±0.16 mg L^-1^) and Chl-a (16.7±0.68 mg m^-3^) in summer were higher than in winter (NO_3_^－^-N, 2.17±0.10 mg L^-1^; COD_Mn_, 2.15±0.10 mg L^-1^; and Chl-a, 9.6±0.33 mg m^-3^).

**
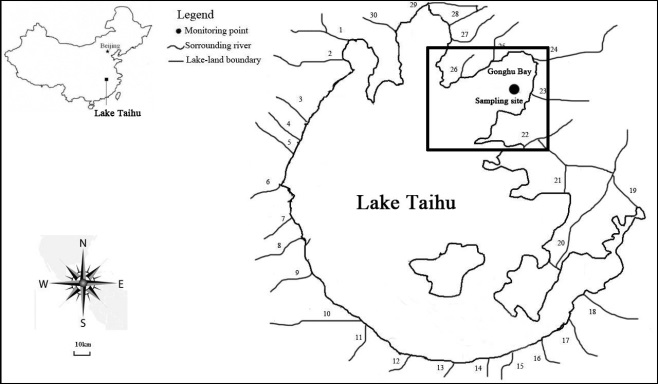

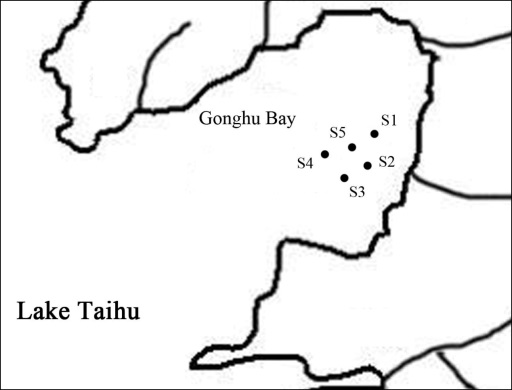
**

## Figure A. Sampling location in Gonghu Bay of Lake Taihu, China.

## Figure B. Images of two species of floating macrophytes *N. peltatum* (a) and *T. natans* (b).


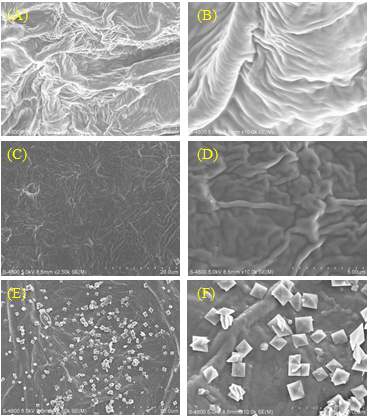


## Figure C. Scanning electron microscope images of the upper leaf surfaces of *N. peltatum* (A and B, summer sample; C and D, winter sample) and *T. natans* (E and F, summer sample). A, C and E, 2500x; B, D and F, 10,000x.


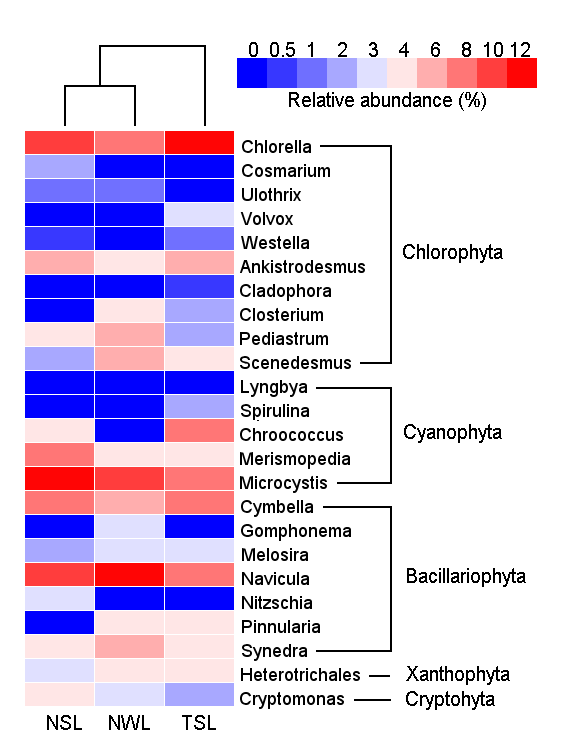


## Figure D. Heat map and cluster analysis of epiphytic algae community (genus/phylum) attached to the leaves of *N. peltatum* in summer (NSL), *N. peltatum* in winter (NWL) and *T. natans* in summer (TSL).


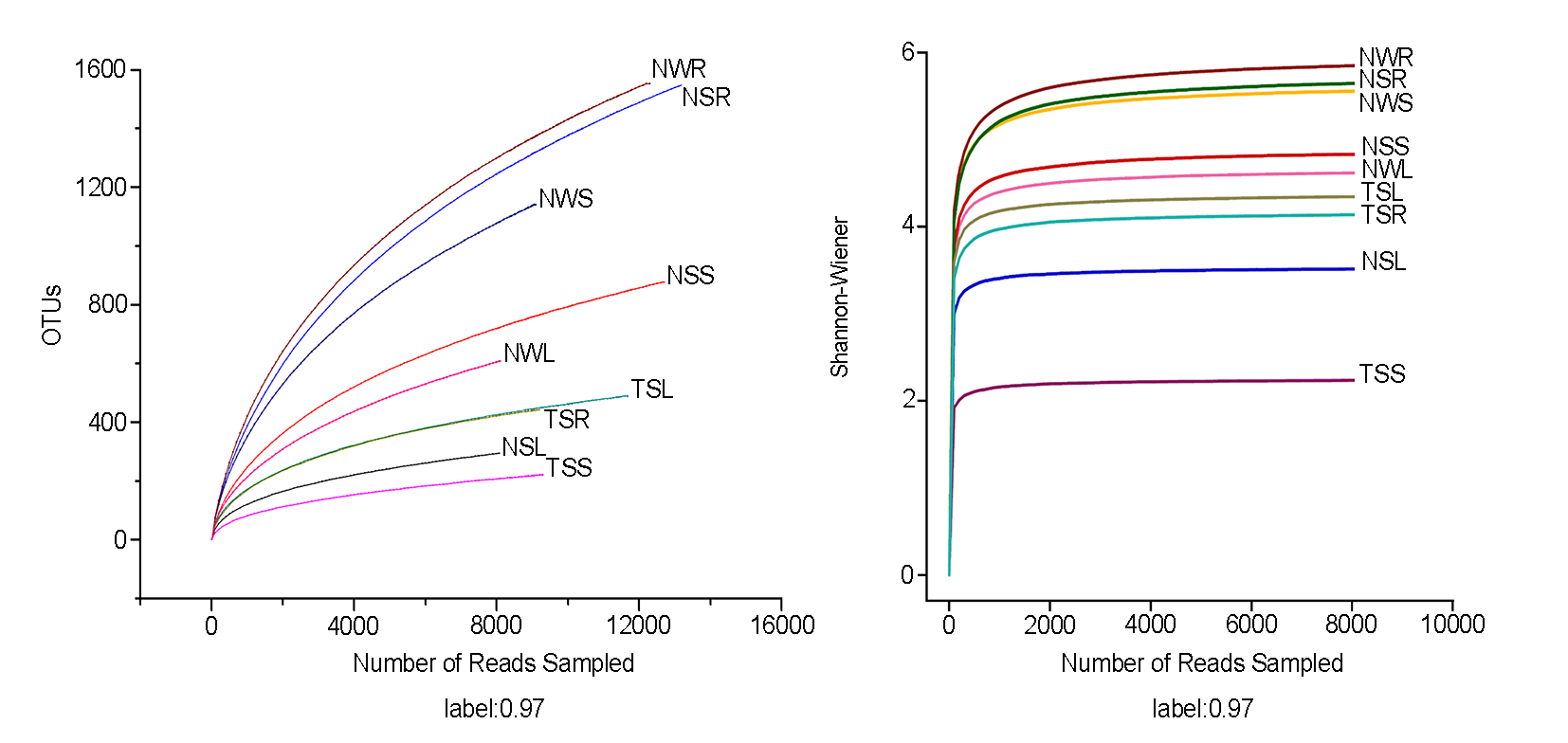


**Figure E. Rarefaction Curve of OTUs (A) and Shannon-Wiener index (B) bases on pyrosequencing reads of 16S rRNA gene from all samples.** NSL, NSS and NSR, biofilms from the leaves, stems and roots of *N. peltatum* in summer, espectively; NWL, NWS and NWR, biofilms from the leaves, stems and roots of *N. peltatum* in winter, respectively; TSL, TSS and TSR, biofilms from the leaves, stems and roots of *T. natans* in summer, respectively.

## Table A. Water quality parameters of sampling sites in Gonghu Bay of Lake Taihu (mg L^-1^).

| **Month** | **TP** | **TN** | **NO_3_^-^-N** | **NO_2_^-^-N** | **NH_4_^+^-N** | **COD_Mn_** | **Chl-a^*^** | **DO** |
| --- | --- | --- | --- | --- | --- | --- | --- | --- |
| **August** | 0.13±0.01 | 3.83±0.18 | 2.79±0.13 | 0.05±0.00 | 0.82±0.04 | 3.56±0.16 | 16.7±0.68 | 8.22±1.4 |
| **December** | 0.17±0.01 | 4.52±0.21 | 2.17±0.10 | 0.07±0.00 | 1.56±0.08 | 2.15±0.10 | 9.6±0.33 | 6.7±1.21 |

*mg m^-3^

**Table B. Sequence of primers used in the present study.**

| **Gene** | **Primer (f-forward;**  **r-reverse)** | **Sequence (5'-3')** | **Reference** |
| --- | --- | --- | --- |
| ***nos*Z** | ***nos*Z_f** | AGAACGACCAGCTGATCGACA | (Ji et al. 2012) |
|  | ***nos*Z_r** | TCCATGGTGACGCCGTGGTTG |  |
| ***qnor*B** | ***qnor*B_f** | GGNCAYCARGGNTAYGA |  |
|  | ***qnor*B_r** | ACCCANAGRTGNACNACCCACCA |  |
| ***Nir*S** | ***Nir*S_f** | GTSAACGTSAAGGARACSGG | (Throback et al 2004) |
|  | ***Nir*S_r** | GASTTCGGRTGSGTCTTGA |  |
| ***nir*K** | ***nir*K_f** | GGMATGGTKCCSTGGCA | (Braker et al 1998) |
|  | ***nir*K_r** | GCCTCGATCAGRTTRTGGTT |  |
| ***cnor*B** | ***cnor*B_f** | GACAAGNNNTACTGGTGGT | (Braker et al 2003) |
|  | ***cnor*B_r** | GAANCCCCANACNCCNGC |  |
| ***nar*G** | ***nar*G_f** | CAYGGNGTNAAYTGYACNGG | (Gregory et al 2000) |
|  | ***nar*G_r** | MGNGGNTGYCCNMGNGGNGC |  |
| ***nap*A** | ***nap*A_f** | GCNCCNTGYMGNTTYTGYGG | (Flanagan et al. 1999) |
|  | ***nap*A_r** | DATNGGRTGCATYTCNGCCATRT |  |

**Table C. Composition of epiphytic algae from the leaves of aquatic plants.**

| **Phylum** | **Genus** | **Percentage** | | |
| --- | --- | --- | --- | --- |
|  |  | **NSL** | **NWL** | **TSL** |
| ***Chlorophyta*** | ***Scenedesmus*** | 2.84 | 6.87 | 5.57 |
|  | ***Chlorella*** | 11.88 | 9.19 | 12.68 |
|  | ***Ankistrodesmus*** | 7.49 | 5.79 | 6.89 |
|  | ***Pediastrum*** | 5.89 | 7.15 | 1.99 |
|  | ***Cosmarium*** | 2.78 | \ | \ |
|  | ***Closterium*** | \ | 4.74 | 2.78 |
|  | ***Ulothrix*** | 1.78 | 1.98 | \ |
|  | ***Tetraedron*** | \ | \ | \ |
|  | ***Westella*** | 0.77 | \ | 1.79 |
|  | ***Volvox*** | \ | \ | 3.29 |
|  | ***Cladophora*** | \ | \ | 0.76 |
| ***Cyanophyta*** | ***Merismopedia*** | 9.72 | 4.65 | 5.71 |
|  | ***Chroococcus*** | 4.68 | \ | 8.93 |
|  | ***Lyngbya*** | \ | \ | 0.28 |
|  | ***Microcystis*** | 13.82 | 10.82 | 9.01 |
|  | ***Spirulina*** | \ | \ | 2.90 |
| ***Bacillariophyta*** | ***Navicula*** | 10.89 | 13.09 | 8.68 |
|  | ***Synedra*** | 5.19 | 7.28 | 5.89 |
|  | ***Cymbella*** | 8.64 | 6.62 | 8.92 |
|  | ***Melosira*** | 2.01 | 3.16 | 3.76 |
|  | ***Pinnularia*** | \ | 5.70 | 4.70 |
|  | ***Nitzschia*** | 3.09 | \ | \ |
|  | ***Gomphonema*** | \ | 3.50 | \ |
| ***Xanthophyta*** | ***Heterotrichales*** | 3.86 | 5.87 | 4.22 |
| ***Cryptophyta*** | ***Cryptomonas*** | 4.67 | 3.58 | 2.24 |

NSL, biofilms from the leaves of *N. peltatum* in summer; NWL, biofilms from the leaves of *N. peltatum* in winter; TSL, biofilms from the leaves of *T. natans* in summer.

### Table D. Summary sequences and Alpha-diversity indexes at 97%.

| **Samples** | **Valid reads** | **Filtered reads** | **Normalized**  **reads** | **OTUs^*^** | **Average**  **length** | **Ace** | **Chao** | **Shannon** | **Coverage** |
| --- | --- | --- | --- | --- | --- | --- | --- | --- | --- |
| **NSL** | 10650 | 8389 | 8023 | 294(3.50%) | 430.25 | 588.4 | 472.5 | 3.47 | 0.99 |
| **NSS** | 15056 | 13339 | 8023 | 878(6.58%) | 422.66 | 1351.88 | 1398.84 | 4.84 | 0.97 |
| **NSR** | 17363 | 15922 | 8023 | 1620(10.17%) | 431.43 | 2404.49 | 2335.68 | 5.69 | 0.96 |
| **NWL** | 14263 | 12346 | 8023 | 606(4.91%) | 427.68 | 1270.24 | 962.24 | 4.62 | 0.97 |
| **NWS** | 10685 | 9506 | 8023 | 1146(12.06%) | 435.18 | 2301.69 | 1909.03 | 5.58 | 0.94 |
| **NWR** | 10032 | 9366 | 8023 | 1551(16.56%) | 426.2 | 2307.15 | 2394.36 | 5.94 | 0.95 |
| **TSL** | 10848 | 9884 | 8023 | 492(4.98%) | 432.95 | 844.98 | 716.38 | 4.38 | 0.98 |
| **TSS** | 15666 | 13829 | 8023 | 221(1.60%) | 432.95 | 500.93 | 400.08 | 2.24 | 0.99 |
| **TSR** | 10622 | 8993 | 8023 | 447(4.97%) | 429.78 | 627.82 | 621.44 | 4.13 | 0.98 |

*Percentages in blankets were calculated as 100%*OTUs/filtered reads. NSL, NSS and NSR, biofilms from the leaves, stems and roots of *N. peltatum* in summer, respectively; NWL, NWS and NWR, biofilms from the leaves, stems and roots of *N. peltatum* in winter, respectively; TSL, TSS and TSR, biofilms from the leaves, stems and roots of *T. natans* in summer, respectively.

### Table E. Abundance of phyla and classes in each sample (%).

| **Phylum** | | | **Class** | **NSL** | **NSS** | **NSR** | **NWL** | **NWS** | **NWR** | **TSL** | **TSS** | **TSR** |
| --- | --- | --- | --- | --- | --- | --- | --- | --- | --- | --- | --- | --- |
| ***Acidobacteria*** | | | -- | 0.26 | 4.40 | 9.90 | 1.36 | 4.03 | 4.67 | 0.32 | 0.25 | 2.96 |
| ***Actinobacteria*** | | | -- | 0.02 | 0.92 | 0.94 | 0.61 | 0.89 | 1.02 | 0.19 | 0.13 | 0.78 |
| ***Armatimonadetes*** | | | -- | 0.04 | 1.35 | 0.88 | 0.18 | 0.32 | 0.72 | 0.26 | 0.09 | 1.08 |
| ***BD1-5*** | | | -- | \ | 0.02 | 0.01 | 0.12 | 0.07 | 0.02 | \ | \ | \ |
| ***BHI80-139*** | | | -- | \ | \ | \ | \ | \ | 0.04 | \ | \ | \ |
| ***Bacteria (Unclassified)*** | | | -- | 0.14 | 0.21 | 1.42 | 2.76 | 1.75 | 1.05 | 0.55 | 0.06 | 0.25 |
| ***Bacteroidetes*** | | | -- | 9.69 | 17.21 | 3.93 | 30.16 | 21.79 | 25.58 | 9.32 | 8.10 | 6.34 |
| ***Candidate_division_BRC1*** | | | -- | 0.04 | 0.10 | 0.32 | 0.11 | 0.36 | 0.49 | 0.03 | 0.02 | 0.03 |
| ***Candidate_division_KB1*** | | | -- | \ | \ | \ | \ | \ | 0.01 | \ | \ | \ |
| ***Candidate_division_OD1*** | | | -- | \ | \ | 0.01 | 0.05 | 0.02 | 0.06 | \ | \ | \ |
| ***Candidate_division_OP8*** | | | -- | 0.05 | 0.29 | 0.77 | 0.26 | 1.13 | 0.60 | \ | \ | 0.01 |
| ***Candidate_division_SR1*** | | | -- | 0.01 | 0.07 | 0.03 | 0.39 | 0.31 | 0.32 | \ | 0.01 | 0.17 |
| ***Candidate_division_TM7*** | | | -- | 0.07 | 0.67 | 0.36 | 0.02 | 0.04 | 0.25 | 0.07 | 0.10 | 1.02 |
| ***Candidate_division_WS3*** | | | -- | 0.01 | 0.08 | 0.96 | 0.32 | 1.72 | 1.11 | \ | \ | 0.01 |
| ***Chlamydiae*** | | | -- | \ | \ | \ | \ | 0.01 | 0.01 | \ | \ | \ |
| ***Chlorobi*** | | | -- | \ | 0.09 | 0.41 | 0.06 | 0.31 | 0.47 | 0.01 | \ | 0.03 |
| ***Chloroflexi*** | | | -- | 0.57 | 12.06 | 6.69 | 1.02 | 3.43 | 4.83 | 1.80 | 1.31 | 23.87 |
| ***Cyanobacteria*** | | | -- | \ | 0.04 | 0.03 | 0.85 | 2.76 | 2.33 | 0.01 | 0.01 | 0.01 |
| ***Deferribacteres*** | | | -- | \ | \ | 0.04 | \ | 0.02 | \ | \ | \ | \ |
| ***Deinococcus-Thermus*** | | | -- | 0.01 | 0.32 | 0.26 | 0.01 | 0.08 | 0.05 | 0.02 | 0.02 | 0.30 |
| ***Elusimicrobia*** | | | -- | \ | \ | \ | 0.01 | 0.04 | \ | \ | \ | \ |
| ***Fibrobacteres*** | | | -- | \ | \ | 0.09 | 0.01 | \ | 0.01 | \ | \ | \ |
| ***Firmicutes*** | | | -- | 13.32 | 6.37 | 4.94 | 0.02 | 0.24 | 0.08 | 12.54 | 8.59 | 25.71 |
| ***Fusobacteria*** | | | -- | 0.21 | 0.02 | 0.01 | \ | \ | \ | 0.04 | 0.01 | \ |
| ***GOUTA4*** | | | -- | \ | \ | \ | \ | 0.01 | \ | \ | \ | \ |
| ***Gemmatimonadetes*** | | | -- | \ | 0.05 | 0.18 | 0.25 | 0.33 | 0.70 | 0.05 | \ | \ |
| ***NPL-UPA2*** | | | -- | \ | 0.02 | 0.02 | \ | \ | 0.07 | \ | \ | \ |
| ***Nitrospirae*** | | | -- | 0.01 | 0.09 | 0.98 | 0.47 | 1.61 | 1.42 | \ | \ | 0.01 |
| ***Planctomycetes*** | | | -- | \ | 0.96 | 0.18 | 0.04 | 0.12 | 0.13 | 0.06 | 0.02 | 0.15 |
| ***Proteobacteria*** | | | *Alphaproteobacteria* | 13.48 | 39.74 | 22.15 | 13.28 | 10.27 | 8.26 | 30.70 | 6.95 | 18.56 |
| -- | | | *Betaproteobacteria* | 5.18 | 3.43 | 13.64 | 34.33 | 23.67 | 20.91 | 12.15 | 0.83 | 2.19 |
| -- | | *Gammaproteobacteria* | | 56.34 | 8.29 | 23.18 | 8.97 | 12.79 | 8.12 | 29.69 | 72.95 | 13.45 |
| -- | | | *Deltaproteobacteria* | 0.05 | 0.63 | 4.05 | 1.47 | 4.78 | 4.20 | 0.30 | 0.06 | 0.04 |
| -- | | *Epsilonproteobacteria* | | 0.02 | \ | 0.19 | 0.10 | 0.04 | 0.06 | \ | 0.01 | \ |
| -- | *Proteobacteria_unclassified* | | | 0.06 | 0.07 | 0.01 | 0.01 | 0.02 | 0.02 | 0.27 | 0.02 | 0.03 |
| -- | | | *Sub-total* | 75.15 | 52.41 | 63.27 | 58.19 | 51.57 | 41.59 | 73.12 | 80.83 | 34.54 |
| ***SHA-109*** | | | -- | 0.01 | 0.03 | 0.03 | 0.02 | 0.04 | 0.07 | \ | \ | \ |
| ***SM2F11*** | | | -- | \ | 0.03 | \ | \ | 0.01 | 0.01 | \ | \ | 0.01 |
| ***Spirochaetae*** | | | -- | \ | 0.02 | 0.39 | 0.02 | 0.32 | 0.34 | \ | \ | \ |
| ***TA06*** | | | -- | 0.02 | 0.01 | 0.07 | \ | 0.15 | 0.10 | \ | \ | \ |
| ***TM6*** | | | -- | \ | 0.02 | 0.01 | \ | \ | \ | \ | \ | \ |
| ***Thermotogae*** | | | -- | \ | \ | 0.01 | \ | 0.01 | 0.01 | \ | \ | \ |
| ***Verrucomicrobia*** | | | -- | 0.36 | 2.10 | 2.76 | 2.66 | 6.48 | 11.82 | 1.63 | 0.44 | 2.70 |
| ***WCHB1-60*** | | | -- | \ | 0.05 | 0.10 | 0.01 | \ | 0.07 | \ | \ | \ |

### NSL, NSS and NSR, biofilms from the leaves, stems and roots of *N. peltatum* in summer, respectively; NWL, NWS and NWR, biofilms from the leaves, stems and roots of *N. peltatum* in winter, respectively; TSL, TSS and TSR, biofilms from the leaves, stems and roots of *T. natans* in summer, respectively.

### Table F. Abundance of dominant orders (abundance >1%) in each sample (%).

| **Order** | **NSL** | | **NSS** | | **NSR** | | **NWL** | | **NWS** | | | **NWR** | | **TSL** | | **TSS** | | **TSR** | |
| --- | --- | --- | --- | --- | --- | --- | --- | --- | --- | --- | --- | --- | --- | --- | --- | --- | --- | --- | --- |
| ***Aeromonadales*** | | 2.24 | | 0.04 | | 2.92 | | 0.01 | | 0.03 | 0.1 | | 0.16 | | \ | | 0.08 | |  |
| ***Anaerolineales*** | | 0.11 | | 0.41 | | 1.68 | | 0.11 | | 0.87 | 0.57 | | 0.03 | | \ | | 0.04 | |  |
| ***Bacillales*** | | 3.27 | | 6.04 | | 3.32 | | 0.01 | | 0.03 | 0.02 | | 12.25 | | 8.56 | | 24.4 | |  |
| ***Bacteroidales*** | | 2.24 | | \ | | 0.19 | | 0.01 | | 0.13 | 0.08 | | 0.23 | | 0.01 | | \ | |  |
| ***Burkholderiales*** | | 2.72 | | 1.9 | | 4.64 | | 22.63 | | 14.49 | 16.42 | | 10.59 | | 0.56 | | 1.59 | |  |
| ***Caldilineales*** | | 0.22 | | 10.14 | | 1.94 | | 0.09 | | 0.9 | 2.97 | | 0.78 | | 0.96 | | 23.43 | |  |
| ***Candidate_division_OP8_norank*** | | 0.05 | | 0.29 | | 0.77 | | 0.26 | | 1.13 | 0.6 | | \ | | \ | | 0.01 | |  |
| ***Candidate_division_WS3_norank*** | | 0.01 | | 0.08 | | 0.96 | | 0.32 | | 1.72 | 1.11 | | \ | | \ | | 0.01 | |  |
| ***Caulobacterales*** | | 1.33 | | 1.14 | | 1.21 | | 0.13 | | 0.08 | 0.38 | | 3.68 | | 0.86 | | 0.28 | |  |
| ***Chloroflexi_uncultured*** | | \ | | 0.14 | | 1.13 | | 0.07 | | 0.43 | 0.37 | | 0.02 | | \ | | 0.03 | |  |
| ***Chromatiales*** | | 0.1 | | 0.09 | | 0.43 | | 1.1 | | 2.1 | 0.84 | | 0.09 | | \ | | \ | |  |
| ***Chthoniobacterales*** | | 0.1 | | 1.8 | | 1.79 | | 1.03 | | 1.33 | 1.5 | | 1.55 | | 0.39 | | 2.36 | |  |
| ***Clostridiales*** | | 4.58 | | 0.05 | | 0.99 | | 0.01 | | 0.14 | 0.02 | | 0.02 | | 0.02 | | 1.26 | |  |
| ***Cyanobacteria_norank*** | | \ | | 0.02 | | 0.01 | | 0.81 | | 2.67 | 2.04 | | 0.01 | | 0.01 | | \ | |  |
| ***Cytophagales*** | | 0.46 | | 0.77 | | 0.35 | | 3.45 | | 2.26 | 4.7 | | 0.64 | | 0.03 | | 0.08 | |  |
| ***Desulfobacterales*** | | \ | | 0.29 | | 2.03 | | 0.59 | | 2.65 | 1.7 | | 0.03 | | \ | | \ | |  |
| ***Enterobacteriales*** | | 47.72 | | 3.95 | | 11.97 | | 0.01 | | 0.28 | 0.07 | | 15.81 | | 11.28 | | 5.23 | |  |
| ***Flavobacteriales*** | | 6.76 | | 12.66 | | 1.61 | | 24.68 | | 15.94 | 14.96 | | 6.84 | | 7.81 | | 3.21 | |  |
| ***Methylococcales*** | | \ | | 0.14 | | 0.14 | | 0.02 | | 1.57 | 0.15 | | \ | | \ | | \ | |  |
| ***Methylophilales*** | | 0.3 | | 0.75 | | 0.25 | | 11.48 | | 8.38 | 3.17 | | 0.63 | | 0.05 | | 0.21 | |  |
| ***Neisseriales*** | | 1.38 | | 0.12 | | 4.57 | | \ | | 0.04 | 0.07 | | 0.15 | | 0.03 | | 0.1 | |  |
| ***Nitrospira_norank*** | | 0.01 | | 0.09 | | 0.98 | | 0.47 | | 1.61 | 1.42 | | \ | | \ | | 0.01 | |  |
| ***OPB35_soil_group_norank*** | | 0.19 | | 0.13 | | 0.54 | | 0.27 | | 1.02 | 2.82 | | 0.01 | | 0.02 | | 0.01 | |  |
| ***Pseudomonadales*** | | 5.95 | | 1.17 | | 2.63 | | 5.85 | | 5.13 | 1.76 | | 12.43 | | 61.47 | | 7.15 | |  |
| ***Rhizobiales*** | | 0.78 | | 4.95 | | 2.26 | | 1.42 | | 0.78 | 1.32 | | 8.93 | | 1.95 | | 3.77 | |  |
| ***Rhodobacterales*** | | 0.43 | | 7.39 | | 1.8 | | 3.85 | | 2.89 | 2.43 | | 6.02 | | 1.08 | | 6.11 | |  |
| ***Rhodocyclales*** | | 0.72 | | 0.28 | | 2.43 | | 0.05 | | 0.21 | 0.19 | | 0.75 | | 0.17 | | 0.07 | |  |
| ***Rhodospirillales*** | | 5.53 | | 0.66 | | 1.74 | | 0.02 | | 0.1 | 0.43 | | 0.62 | | 0.06 | | 0.52 | |  |
| ***Rickettsiales*** | | 0.11 | | 0.46 | | 0.07 | | 1.07 | | 1.19 | 0.62 | | 0.47 | | 0.09 | | 0.08 | |  |
| ***S-BQ2-57_soil_group_norank*** | | \ | | \ | | 0.12 | | 0.43 | | 0.94 | 1.35 | | \ | | \ | | \ | |  |
| ***Sphingomonadales*** | | 4.63 | | 24.88 | | 14.98 | | 6.68 | | 5.17 | 2.78 | | 10.4 | | 2.83 | | 7.67 | |  |
| ***Subgroup_4*** | | 0.09 | | 2.21 | | 0.49 | | 0.04 | | 0.06 | 0.33 | | 0.15 | | 0.22 | | 2.44 | |  |
| ***Subgroup_6*** | | 0.12 | | 1.68 | | 5.65 | | 0.69 | | 2.43 | 1.97 | | 0.14 | | 0.02 | | 0.48 | |  |
| ***Verrucomicrobiales*** | | \ | | 0.16 | | 0.32 | | 0.81 | | 2.64 | 5.81 | | 0.07 | | 0.03 | | 0.32 | |  |
| ***Xanthomonadales*** | | 0.31 | | 2.8 | | 4.26 | | 1.66 | | 2.73 | 4.23 | | 1.04 | | 0.18 | | 0.9 | |  |

### NSL, NSS and NSR, biofilms collected from leaves, stems and roots of *N. peltatum* in summer, respectively; NWL, NWS and NWR, biofilms collected from leaves, stems and roots of *N. peltatum* in winter, respectively; TSL, TSS and TSR, biofilms collected from leaves, stems and roots of *T. natans* in summer, respectively.

### Table G. Abundance of top 10 families (figure in box) in each sample (%).

| **Family** | ***N. peltatum* in summer** | | | ***N. peltatum* in winter** | | | ***T. natans* in summer** | | |
| --- | --- | --- | --- | --- | --- | --- | --- | --- | --- |
|  | **NSL** | **NSS** | **NSR** | **NWL** | **NWS** | **NWR** | **TSL** | **TSS** | **TSR** |
| ***Aeromonadaceae*** | 2.24 | 0.04 | 2.92 | 0.01 | 0.03 | 0.10 | 0.16 | \ | 0.08 |
| ***Caldilineaceae*** | 0.22 | 10.14 | 1.94 | 0.09 | 0.90 | 2.97 | 0.78 | 0.96 | 23.43 |
| ***Caulobacteraceae*** | 1.31 | 0.88 | 0.98 | 0.04 | 0.03 | 0.21 | 3.65 | 0.86 | 0.25 |
| ***Chitinophagaceae*** | 0.15 | 3.31 | 0.99 | 0.21 | 0.62 | 1.00 | 1.48 | 0.23 | 2.59 |
| ***Clostridiaceae_1*** | 4.33 | 0.02 | 0.37 | \ | 0.06 | \ | 0.02 | \ | 0.03 |
| ***Comamonadaceae*** | 2.68 | 1.32 | 3.56 | 14.58 | 7.13 | 13.46 | 10.50 | 0.41 | 1.27 |
| ***Cyanobacteria_norank*** | \ | 0.02 | 0.01 | 0.81 | 2.67 | 2.04 | 0.01 | 0.01 | \ |
| ***Cytophagaceae*** | 0.46 | 0.74 | 0.33 | 3.15 | 1.65 | 4.27 | 0.63 | 0.03 | 0.08 |
| ***Enterobacteriaceae*** | 47.72 | 3.95 | 11.97 | 0.01 | 0.28 | 0.07 | 15.81 | 11.28 | 5.23 |
| ***Erythrobacteraceae*** | 0.46 | 7.10 | 2.03 | 0.63 | 0.45 | 0.26 | 1.98 | 0.35 | 1.83 |
| ***Family_XII*** | 1.12 | 5.93 | 3.27 | 0.01 | 0.02 | 0.02 | 11.71 | 7.71 | 20.50 |
| ***Flavobacteriaceae*** | 6.76 | 12.61 | 1.42 | 24.02 | 15.55 | 14.42 | 6.76 | 7.80 | 3.13 |
| ***Methylophilaceae*** | 0.30 | 0.75 | 0.25 | 11.48 | 8.38 | 3.17 | 0.63 | 0.05 | 0.21 |
| ***Moraxellaceae*** | 0.06 | 0.97 | 1.26 | 1.86 | 0.81 | 0.20 | 10.95 | 61.01 | 6.92 |
| ***Neisseriaceae*** | 1.38 | 0.12 | 4.57 | \ | 0.04 | 0.07 | 0.15 | 0.03 | 0.10 |
| ***OPB35_soil_group_norank*** | 0.19 | 0.13 | 0.54 | 0.27 | 1.02 | 2.82 | 0.01 | 0.02 | 0.01 |
| ***Oxalobacteraceae*** | \ | 0.01 | 0.01 | 7.88 | 7.04 | 2.35 | 0.01 | 0.02 | \ |
| ***Planococcaceae*** | 0.84 | 0.02 | 0.23 | \ | 0.01 | \ | 0.01 | 0.18 | 3.66 |
| ***Pseudomonadaceae*** | 5.89 | 0.20 | 1.37 | 3.98 | 4.31 | 1.56 | 1.48 | 0.46 | 0.23 |
| ***Rhizobiaceae*** | 0.51 | 1.85 | 0.87 | 1.29 | 0.21 | 0.29 | 7.14 | 1.40 | 1.12 |
| ***Rhodobacteraceae*** | 0.43 | 7.39 | 2.43 | 3.85 | 2.89 | 2.43 | 6.02 | 1.08 | 6.11 |
| ***Rhodospirillaceae*** | 4.39 | 0.17 | 1.13 | \ | \ | 0.10 | 0.29 | 0.01 | \ |
| ***Sphingomonadaceae*** | 3.61 | 11.24 | 6.99 | 2.73 | 1.10 | 1.13 | 4.93 | 0.82 | 3.41 |
| ***Unclassified Sphingomonadales*** | 0.56 | 6.32 | 5.83 | 3.24 | 3.37 | 1.31 | 3.44 | 1.66 | 2.36 |
| ***Subgroup_6_norank*** | 0.12 | 1.68 | 5.65 | 0.69 | 2.43 | 1.97 | 0.14 | 0.02 | 0.48 |
| ***Veillonellaceae*** | 4.89 | 0.26 | 0.35 | \ | \ | 0.04 | 0.19 | 0.01 | 0.03 |
| ***Verrucomicrobiaceae*** | \ | 0.16 | 0.32 | 0.81 | 2.63 | 5.81 | 0.07 | 0.03 | 0.32 |
| ***Xanthomonadaceae*** | 0.31 | 1.89 | 2.40 | 1.26 | 1.88 | 3.36 | 0.99 | 0.18 | 0.66 |

### NSL, NSS and NSR, biofilms collected from leaves, stems and roots of N. peltatum in summer, respectively; NWL, NWS and NWR, biofilms collected from leaves, stems and roots of N. peltatum in winter, respectively; TSL, TSS and TSR, biofilms collected from leaves, stems and roots of T. natans in summer, respectively.

### Table H. Numbers of detected genera in nine samples.

| **Samples** | **NSL** | **NSS** | **NSR** | **NWL** | **NWS** | **NWR** | **TSL** | **TSS** | **TSR** | **Total** |
| --- | --- | --- | --- | --- | --- | --- | --- | --- | --- | --- |
| **Genus numbers** | 167 | 318 | 474 | 239 | 362 | 419 | 222 | 129 | 199 | 677 |

NSL, NSS and NSR, biofilms from the leaves, stems and roots of *N. peltatum* in summer, respectively; NWL, NWS and NWR, biofilms from the leaves, stems and roots of *N. peltatum* in winter, respectively; TSL, TSS and TSR, biofilms from the leaves, stems and roots of *T. natans* in summer, respectively.

### Table I. Results of two-way ANOVA analysis on richness of denitrifying genes.

| **Group** | **Factor** | | ***napA*** | ***narG*** | ***qnorB*** | ***nirS*** | ***nirK*** | ***cnorB*** | ***nosZ*** |
| --- | --- | --- | --- | --- | --- | --- | --- | --- | --- |
| **Samples from**  ***N. peltatum*** | **season** | ***F*** | 59.78 | 1248 | 3807 | 1136 | 29.74 | 1.366 | 1162 |
|  |  | ***Sig.*** | 0.000 | 0.000 | 0.000 | 0.000 | 0.000 | 0.265 | 0.000 |
|  | **plant organ** | ***F*** | 1545 | 1092 | 613.7 | 522.2 | 71.91 | 4.280 | 840.3 |
|  |  | ***Sig.*** | 0.000 | 0.000 | 0.000 | 0.000 | 0.000 | 0.04 | 0.000 |
|  | **season*plant organ** | ***F*** | 336.8 | 250.2 | 921.1 | 88.22 | 1592 | 160.4 | 18.96 |
|  |  | ***Sig.*** | 0.000 | 0.000 | 0.000 | 0.000 | 0.000 | 0.000 | 0.000 |
| **Samples in summer** | **plant species** | ***F*** | 33.57 | 32.69 | 236.5 | 145.8 | 14.96 | 164.1 | 2263 |
|  |  | ***Sig.*** | 0.000 | 0.000 | 0.000 | 0.000 | 0.002 | 0.000 | 0.000 |
|  | **plant organ** | ***F*** | 836.6 | 175.0 | 571.5 | 331.5 | 426.7 | 1150 | 708.5 |
|  |  | ***Sig.*** | 0.000 | 0.000 | 0.000 | 0.000 | 0.000 | 0.000 | 0.000 |
|  | **plant species* plant organ** | ***F*** | 606.2 | 108.3 | 40.39 | 3488 | 440.0 | 836.6 | 1832 |
|  |  | ***Sig.*** | 0.000 | 0.000 | 0.000 | 0.000 | 0.000 | 0.000 | 0.000 |

# References

Braker, G., Fesefeldt, A., Witzel, K.P. 1998. Development of PCR primer systems for amplification of nitrite reductase genes (nirK and nirS) to detect denitrifying bacteria in environmental samples. *Appl. Environ. Microbiol.* 64(10):3769-3775.

Braker, G., Tiedje, J.M. 2000. Nitric oxide reductase (norB) genes from pure cultures and environmental samples. *Appl. Environ. Microbiol*. 2003;69(6):3476-83.

Flanagan, D.A. and others 1999. Detection of genes for periplasmic nitrate reductase in nitrate respiring bacteria and in community DNA. *Fems Microbiol. Lett.* 1999;177(2):263-70.

Gregory, L.G., Karakas-Sen, A., Richardson, D.J., Spiro, S. 2000. Detection of genes for membrane-bound nitrate reductase in nitrate-respiring bacteria and in community DNA. *Fems Microbiol. Lett.* 183(2):275-279.

Ji, G.D., Zh.i W., Tan. Y.F. 2012. Association of nitrogen micro-cycle functional genes in subsurface wastewater infiltration systems. *Ecol. Eng.* 44:269-277.

Throback, I.N., Enwall, K., Jarvis, A., Hallin, S. 2004. Reassessing PCR primers targeting nirS, nirK and nosZ genes for community surveys of denitrifying bacteria with DGGE. *Fems Microbiol. Ecol.* 49(3):401-417.
